# Supplementary material for: Investigating host-virus interaction mechanism and phylogenetic analysis of viral proteins involved in the pathogenesis
Source: PLoS One. 2021 Dec 16;16(12):e0261497. doi: 10.1371/journal.pone.0261497 (PMC8675761; doi:10.1371/journal.pone.0261497)
Supplement: Attachment — Submitted filename: Rebuttle Letter.docx [file pone.0261497.s001.docx]

**Reviewers’ Comments**

Reviewer #1: This paper attempts to bring and correlation between viral pathogenies with that of viral genome organization. Specifically deciphering the role of viral attachment proteins. This topic is very broad. The first half of the paper contains a very general description of the virus biology of several human pathogens. In the latter half, the authors produce phylogenetic evidence of major viral attachment protein and look for some common ground to relate it the pathogenesis.

1. In my view the paper could have a more organized approach. This manuscript contains lots of facts, general things.

*Response 1: As per the suggestion of the review the authors have revised and updated the structure and language of the manuscript to make it more readable.*

2. The authors need to make it more appealing by detailing the phylogenetic information with that of virus biology cum viral pathogenesis. In a simple way, please answer how the phylogenetic information is important to predict viral pathogenesis.

*Response 2: As suggested, changes have been made in the manuscript to elaborate the role of phylogenetic analysis and pathogenesis.*

3. How does the structural/viral attachment protein genetic make-up predictably results in viral entry, attachment, receptor binding, and providing an opportunity for drug discovery?

*Response 3: This is evident from the literature available and ongoing vaccine development projects worldwide, understanding of host-virus interaction mechanism play a crucial role for the development of effective vaccines and therapeutic interventions. Therefore, we aim to shed light on these processes that help the virus to enter host cell and replicate.*

4. Working with too many viruses at a time is clearly going out of the focus. I recommend that the authors should pick a family of viruses such as flaviviruses, rhabdovirus, coronaviridae, etc., goes to the species level, look at the minute details in viral attachment proteins and establish the link between the host receptor and pathogenies.
More importantly, the introduction and abstract section should highlight specific new findings, insights, or perspectives.

*Response 4: The authors have selected the various based on the prevalence of disease they cause across the globe. Mainly those viruses that have been and are associated with epidemic and pandemic like situations are discussed. As suggested, the manuscript has been revised to eliminate possible grammatical errors.*

5. Grammar and English sentences need thorough checking,

*Response 5: As suggested by the reviewer, the manuscript has been revised and checked for grammatical and spelling mistakes.*

6. Please focus on a specific group of viruses, and look at the species variation, link it to virus biology. Coronavirus should be highlighted.

*Response 6:* *The authors have selected the various based on the prevalence of disease they cause across the globe. Mainly those viruses that have been and are associated with epidemic and pandemic like situations are discussed.*


7. Quality of figures may be improved.

*Response 7: Figures have been improved to match the required quality measures.*

8. Please avoid passive grammars in sentence structure. References should be thoroughly checked.

*Response 8: As per the suggestion of the reviewer, the manuscript has been revised to remove grammatical errors and passive sentence structure as much as possible.*

**Reviewer #2**

In this review article, authors discuss the host-virus interaction mechanisms and the molecular machinery involved in the pathogenesis of the influenza virus, hepatitis virus, human immunodeficiency virus, and coronaviruses. They discuss the genomic organization of these viruses, including the viral proteins that play crucial roles in viral attachment, membrane fusion, cell entry, replication and subsequent release of the mature virion particles in the host environment where they infect. To gain insights into the viral origin's evolutionary perspectives, they have claimed to envisage the phylogeny of viral proteins to understand the changes in the sequence organization of these proteins during evolution for various strains of the viruses.

1. The phylogenetic analysis, which is main highlight of the review according to the authors, seems weak.

*Response 1: As per the suggestion of the reviewer, the section discussing phylogenetic analysis has been updated to make it more elaborate.*

2. Why authors have rely on summarizing the viral pathogenesis almost half of the manuscript.

*Response 2: The rationale behind discussing the pathogenesis and viral proteins involved in pathogenesis was to lay the foundation for highlighting the role of these proteins and to link their significant in the phylogenetic analysis. However, the pathogenesis section has been trimmed to reduce the redundancy.*

3. Out of phylogenetic analysis presented, it is difficult to fetch, what conclusion authors wants to draw.

*Response 3: Phylogenetic analysis has been updated to make it more comprehensible. Our aim was to elaborate the evolutionary changes in the viral proteins over the span of time that play significant role in hurdles in vaccine development since most vaccine and antiviral drugs target proteins involved in cell attachment and fusion.*

4. Till, they will discuss the take home messages of each of the trees and MSA representations, it is difficult to understand what evolutionary significance they provide.

*Response 4: Phylogenetic analysis has been modified for clarity and comprehension.*

5. The manuscript need to express in the introduction/ background section comments on current body of literature, after that, what are the gaps in our understanding of viral molecular evolution. The title of the manuscript is totally a mismatch from the contents.

*Response 5: As per the suggestion of the reviewer, introduction section of the manuscript has been updated.*

6. Language editing is required to improve the quality of manuscript.

*Response 6: In the light of reviewer’s comment, the manuscript has been revised thoroughly to improve the quality of language and eliminate grammatical and spelling mistakes.*
